# Impact of Small Vessel Disease Progression on Long-term Cognitive and Functional Changes After Stroke

**DOI:** 10.1212/WNL.0000000000200005

**Published:** 2022-04-05

**Authors:** Una Clancy, Stephen D.J. Makin, Caroline A. McHutchison, Vera Cvoro, Francesca M. Chappell, Maria del C. Valdés Hernández, Eleni Sakka, Fergus Doubal, Joanna M. Wardlaw

**Affiliations:** From the Centre for Clinical Brain Sciences (U.C., S.D.J.M., C.A.M., V.C., F.M.C., M.d.C.V.H., E.S., F.D., J.M.W.), Edinburgh Imaging and the UK Dementia Research Institute, University of Edinburgh; and Centre for Rural Health (S.D.J.M.), Institute of Applied Health Sciences, University of Aberdeen, UK.

## Abstract

**Background and Objectives:**

The severity of white matter hyperintensities (WMH) at presentation with stroke is associated with poststroke dementia and dependency. However, WMH can decrease or increase after stroke; prediction of cognitive decline is imprecise; and there are few data assessing longitudinal interrelationships among changing WMH, cognition, and function after stroke, despite the clinical importance.

**Methods:**

We recruited patients within 3 months of a minor ischemic stroke, defined as NIH Stroke Scale (NIHSS) score <8 and not expected to result in a modified Rankin Scale (mRS) score >2. Participants repeated MRI at 1 year and cognitive and mRS assessments at 1 and 3 years. We ran longitudinal mixed-effects models assessing change in Addenbrooke’s Cognitive Examination–Revised (ACE-R) and mRS scores. For mRS score, we assessed longitudinal WMH volumes (cube root; percentage intracranial volume [ICV]), adjusting for age, NIHSS score, ACE-R, stroke subtype, and time to assessment. For ACE-R score, we additionally adjusted for ICV, mRS, premorbid IQ, and vascular risk factors. We then used a multivariate model to jointly assess changing cognition/mRS score, adjusted for prognostic variables, using all available data.

**Results:**

We recruited 264 patients; mean age was 66.9 (SD 11.8) years; 41.7% were female; and median mRS score was 1 (interquartile range 1–2). One year after stroke, normalized WMH volumes were associated more strongly with 1-year ACE-R score (β = −0.259, 95% CI −0.407 to −0.111 more WMH per 1-point ACE-R decrease, *p* = 0.001) compared to subacute WMH volumes and ACE-R score (β = 0.105, 95% CI −0.265 to 0.054, *p* = 0.195). Three-year mRS score was associated with 3-year ACE-R score (β = −0.272, 95% CI −0.429 to −0.115, *p* = 0.001). Combined change in baseline-1-year jointly assessed ACE-R/mRS scores was associated with fluctuating WMH volumes (*F* = 9.3, *p* = 0.03).

**Discussion:**

After stroke, fluctuating WMH mean that 1-year, but not baseline, WMH volumes are associated strongly with contemporaneous cognitive scores. Covarying longitudinal decline in cognition and independence after stroke, central to dementia diagnosis, is associated with increasing WMH volumes.

Cerebral small vessel disease (SVD) is common in patients with stroke and is a common cause of stroke and vascular dementia.^1^ White matter hyperintensities (WMH), a key feature of SVD, have been shown to decrease and increase in stroke,^2^ cognitively impaired individuals,^3,4^ older community-dwelling adults investigated for neurologic or cognitive symptoms,^5^ and healthy adults (mean age 63 years).^6^ It is not clear how the timing of SVD lesion change relates to the clinical development of stroke and dementia.

To diagnose dementia, evidence of coexisting decline in both cognitive and functional ability is required. After stroke, we usually define functional status according to the degree of poststroke disability measured by the modified Rankin Scale (mRS), that is, the level of independence in carrying out activities of daily living. In particular, mild neurocognitive disorder is distinguished from major neurocognitive disorder by the presence of increasing dependence.^7,8^ This is particularly relevant after stroke because both worsening mRS score and cognitive impairment are common and interrelated 3 years after minor stroke.^9^ However, it is not known whether SVD progression might influence the interaction of these impairments.

In cross-sectional studies, worse WMH at stroke presentation are associated with worse cognition assessed concurrently and long term.^10,11^ However, it is less clear whether longitudinal WMH change predicts coexistent poststroke cognitive or functional impairment. Relatively small MRI studies assessing WMH progression and poststroke cognitive decline have not detected a longitudinal association. However, follow-up sample sizes may have been underpowered, ranging from 30 to 94 participants^12-17^ (systematic search for relevant papers and a summary of their characteristics are given in eTables 1 and 2, links.lww.com/WNL/B796). In addition, we and others showed recently that WMH can decrease and increase long term,^2,5^ and although the reasons are poorly understood, fluctuations in WMH have not been accounted for in longitudinal studies. Although numerous studies have assessed baseline WMH and mRS change after stroke,^10^ we are not aware of any studies that have assessed WMH progression and mRS score change after stroke (eTables 1 and 2) or if WMH progression or severity at specific time points affects variation between both mRS and cognition scores after stroke, despite these measures being collected increasingly by ongoing studies.^18,19^

The present in-depth analysis builds on previous work from the Mild Stroke Study-2^9^ on 1- and 3-year outcomes after stroke that included predictors of cognition and cognition–mRS score relationships^9^ but did not assess longitudinal change incorporating all 3 elements of cognition, mRS score, and WMH.

We aimed to determine whether WMH associations with cognition and mRS score vary at different time points after stroke; whether associations vary according to intraindividual trajectories; whether longitudinal WMH volume change is associated with change in cognition and mRS as a coprimary, covarying endpoint; and whether any associations more strongly drive change in cognition or mRS score.

## Methods

### Participants and Design

**Figure 1:**
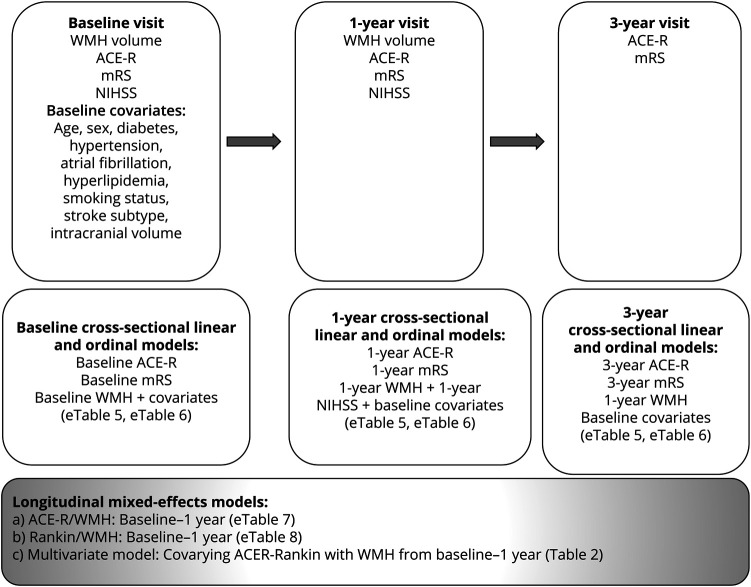
Study and Analysis Flowchart Age is age at recruitment. Diabetes is defined as a previous diagnosis or diagnosed at stroke presentation in accordance with the World Health Organization criteria. Hypertension is defined as a previous diagnosis or blood pressure of ≥140/90 mm Hg at presentation. Hyperlipidemia is defined as a previous diagnosis or cholesterol >5 mmol/L at presentation. Smoking status is defined as self-report of current, ex-smoker <1 year ago, ex-smoker >1 year ago, or never smoker. Stroke subtyping was defined previously.^20^ ICV is defined as outlined in Methods. ACE-R = Addenbrooke's Cognitive Assessment–Revised; mRS = modified Rankin Scale; NIHSS = NIH Stroke Scale; WMH = white matter hyperintensities.

We prospectively recruited 264 patients presenting to stroke services in Edinburgh, UK, with acute minor ischemic stroke. All patients were dementia-free at recruitment and were treated with secondary stroke prevention according to guidelines. Participants had baseline assessments 1 to 3 months after stroke that included MRI, cognitive, and mRS assessments. Participants repeated MRI at 1 year and repeated cognitive and mRS assessments at 1 and 3 years. Figure 1 shows variables collected at each visit.

### Longitudinal Cognitive, mRS Score, and Other Clinical Assessments

Study procedures, including baseline variables and stroke subtyping, have been described in full previously.^2,9,20-22^ Participants underwent the Addenbrooke's Cognitive Examination–Revised version (ACE-R) at all 3 visits. The ACE-R is sensitive to multidomain cognitive impairment after stroke, similar to the Montreal Cognitive Assessment.^23^ We assessed the mRS at all 3 visits. We collected NIH Stroke Scale (NIHSS) score at baseline and 1 year. At baseline, participants completed the National Adult Reading Test (NART), a durable measure of premorbid intelligence.^22^

### Magnetic Resonance Imaging

#### MRI Acquisition

Participants had brain MRI at baseline and were invited to repeat MRI 1 year later, all performed on the same 1.5T scanner (Signa LX; General Electric, Milwaukee, WI). MRI included 3D T1-, T2-, and gradient recalled echo T2*-weighted, fluid-attenuated inversion recovery (FLAIR), and diffusion tensor imaging sequences using a self-shielding gradient and 8-channel phased-array head coil (details described previously).^24^

#### MRI Analysis

We analyzed all images according to the Standards for Reporting Vascular Changes on Euroimaging^25^ using validated computational pipelines and visual assessments. Image analysis methods are outlined in full elsewhere.^24^ In brief, we coregistered structural sequences at both time points using FSL-FLIRT. Total brain WMH volumes were quantified semiautomatically with paired T2-weighted, FLAIR, and T2*-weighted images. WMH were carefully distinguished from the site of all chronic, index, and incident stroke lesions to avoid distorting WMH volume measurements.^26^ This was guided by visual checks of diffusion-weighted, T2-weighted, and FLAIR images in discussion with a neuroradiologist (J.M.W.).^26^

We extracted baseline intracranial volume (ICV) semiautomatically using the T2*-weighted sequence followed by manual correction. All results were checked and manually edited independently by investigators blinded to all clinical information.

### Standard Protocol Approvals, Registrations, and Patient Consents

This study was approved by Lothian Ethics Medical Research Committee (REC 09/81,101/54) and NHS Lothian R&D Office (2009/W/NEU/14). All participants gave written informed consent.

### Statistical Analysis

#### Data Preparation

We used all available data at each time point. To improve model fit, we transformed WMH volumes, calculating the cube root and expressing the result as percent ICV, described in previous analyses.^2^ We also rescaled ICV to ensure model convergence, due to the difference in magnitude vs other variables, dividing by 1,000 to improve model fit. To avoid model overfitting, we calculated a baseline vascular risk factor composite sum score.^27^ This score assigned equal weight to hypertension, hyperlipidemia, diabetes, and smoking history. We created WMH volume change quintiles between baseline and 1 year for display purposes, but we used continuous WMH volume measurements for all statistical analyses. Years since stroke was categorized as 0 (baseline visit was 1–3 months after stroke) vs 1-year visit.

#### Models

Figure 1 describes the cross-sectional and longitudinal data analyses.

First, we assessed whether cross-sectional WMH volume–cognition–mRS score associations are stronger or weaker at the different key time points after stroke. To achieve this, we ran linear models with ACE-R score at each visit and ordinal regression models with mRS score at each visit (Figure 1), all adjusted for key factors that we outline below.

Second, we assessed longitudinal change according to intraindividual trajectories. To do this, we performed mixed-effects analyses. To assess cognitive decline, we used a linear mixed-effects model (lme4,^28^ R, R Foundation for Statistical Computing, Vienna, Austria) with longitudinal change in ACE-R score between baseline and 1 year as the dependent variable. Because data for participants who did not attend follow-up visits or who died after baseline (n = 5 at 1 year, n = 22 at 3 years) are not missing at random, we did not perform multiple imputation or last observation carried forward in accordance with guidance^29,30^; therefore, visits with missing data for such participants were not used. However, when available, baseline and/or 1-year data for these participants were still used in the mixed-effects models. In keeping with previous analyses of poststroke cognitive course,^9^ we adjusted for normalized longitudinal WMH volumes, baseline and 1-year mRS score, time since stroke (baseline vs 1 year), and the following baseline variables: age, NART score, stroke subtype, vascular risk factors, and ICV (to account for original brain size).^25^

To assess worsening mRS score, we performed mixed-effects ordinal regression analysis (mixor,^31^ R) with longitudinal mRS score change between baseline and 1 year (dependent variable). In this model, we adjusted for normalized longitudinal WMH volumes (baseline to 1 year), baseline and 1-year ACE-R score, NIHSS score, time since stroke (baseline vs 1 year), and baseline age and stroke subtype. For participants who died after baseline or 1-year follow-up, we used any available baseline or 1-year data collected when these patients were alive in mixed-effects models. We did not adjust for sex because it appears not to predict long-term independence^32^ or cognitive function^11^ after stroke. For this ordinal analysis, we standardized normalized WMH volumes using *z* scores. This minimizes the parameter scaling issues with ordinal dependent variables that can hinder model convergence.

Last, to assess covarying change in cognition and mRS scores between baseline and 1 year, we created a multivariate mixed-effects model combining cognitive and mRS scores as a coprimary outcome. We fitted baseline and 1-year normalized WMH volumes, NIHSS score, time since stroke (baseline vs 1 year), and the following baseline variables as fixed effects: age, ICV, stroke subtype, and vascular risk factor score. We built the model using ASReml-R version 4.^33^ This analysis included baseline and 1-year data because we collected imaging data at these visits only. We excluded individual participants' time points that did not contain a 1-year WMH volume measurement (n = 68 of 264) or baseline vascular risk factor sum score (n = 2 of 264). For all mixed-effects analyses, we fitted individual participants as random effects. The benefit of this approach is that it assesses intraindividual variation over time. We reported conditional *F* test and *p* values for fixed effects. We chose predictors on the basis of previous research^9^ and did not perform power calculations on existing data as per recommended practice.^34^

### Data Availability

Anonymized data not published within this article can be made available by request from any qualified investigator.

## Results

We collected mRS, imaging, and cognitive data at baseline (mRS score n = 264, imaging n = 264, cognitive n = 157) and 1 year (mRS score n = 264, imaging n = 196, cognitive n = 151) and 3 years (mRS score n = 222, no imaging, cognitive n = 152) after stroke.

Participants who did not attend the 1-year follow-up (n = 68 of 264) were older (mean age 69.9 [SD 13.3] years vs 65.8 [11.1] years), had lower ACE-R scores (mean 83 [SD 9.7] vs 88.8 [7.6]), and had similar WMH volumes (median 13.4 [interquartile range (IQR) 5.0–36.8] mL vs 12.8 [4.4–33.7] mL) and vascular risk factor scores (mean 1.8 [SD 1.0] vs 1.6 [0.8]) at baseline than participants who did attend. Participants who did not attend the 3-year cognitive follow-up (n = 112 of 264) were older at baseline (68.6 [SD 12.5] years vs 65.6 [11.0] years) and had similar baseline ACE-R scores (86.9 [8.6] vs 88.7 [7.8]), higher baseline WMH volumes (median 17.8 [IQR 5.9–40] mL vs 12.3 [3.8–29.4] mL), and similar vascular risk factor scores (mean 1.8 [1.0] vs 1.6 [0.94]) than those who did. Differences are shown in eTable 3. At the 1-year follow-up, 5 of 264 (1.9%) had died. At the 3-year follow-up, 22 of 222 (9.9%) had died.

At baseline, the mean age was 66.9 (SD 11.8) years; 41.7% were female; median mRS score was 1 (IQR 1–2); median NIHSS score was 1 (IQR 0–2); median WMH volume was 13.1 mL (IQR 4.5–34 mL); median WMH as percent ICV was 0.89 (IQR 0.31–2.38); and mean ACE-R score was 88.1 (SD 8.1). Stroke subtype was lacunar in 44.6%. Population characteristics at baseline are shown in Table 1.

**Table 1:**
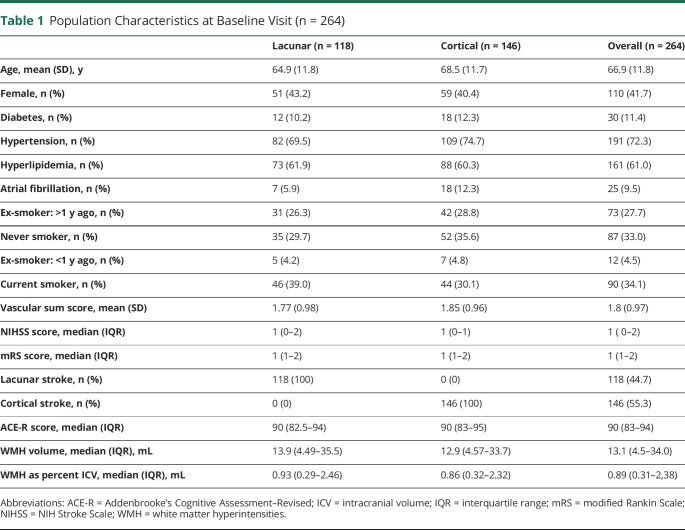
Population Characteristics at Baseline Visit (n = 264)

**Figure 2:**
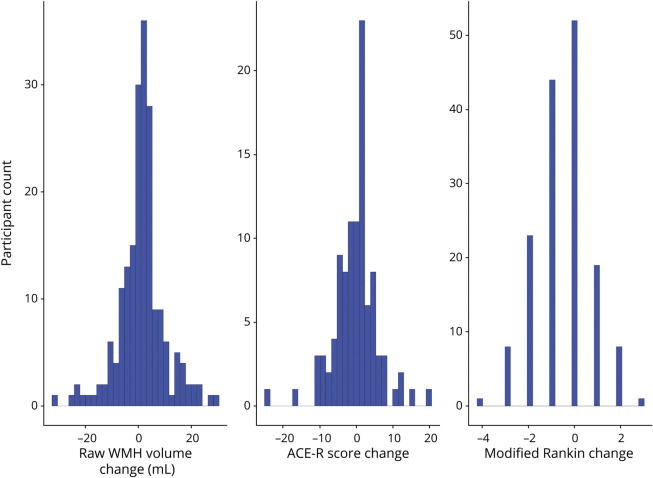
Distribution of Change in WMH Volumes (Baseline–1 Year), ACE-R Scores, and mRS Scores (Baseline–3 Years) ACE-R = Addenbrooke's Cognitive Assessment–Revised; mRS = modified Rankin Scale; WMH = white matter hyperintensities.

**Figure 3:**
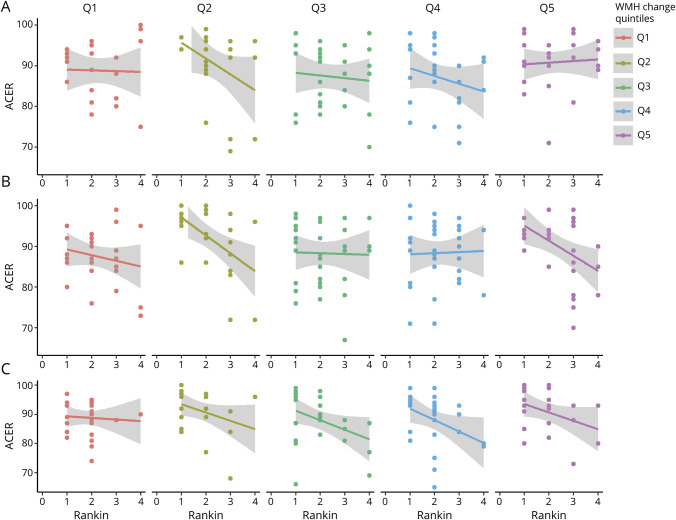
Cross-Sectional Correlations Between ACE-R and mRS Scores at Each Time Point (A) Baseline, (B) 1-year, and (C) 3-year visits, divided into panels by extent of baseline–1-year white matter hyperintensity (WMH) change. Quintile (Q) 5 = greatest WMH increase; Q1 = greatest WMH reduction. Note that participants with modified Rankin Scale (mRS) scores of 5 or 6 did not complete Addenbrooke's Cognitive Assessment–Revised (ACE-R).

Over follow-up, the mean difference in WMH volumes between baseline and 1 year was 1.31 (SD 8.68) mL with maximum 29.1 mL growth and maximum 31.9 mL shrinkage. The mean change in ACE-R scores from baseline to 3 years was −0.17 (SD 5.93), with a maximum increase of 20 points and maximum decrease of 24 points. The median change in mRS score from baseline to 3 years was 0 (IQR −1 to 0), with a maximum increase of 3 points and maximum decrease of 4 points. Figure 2 shows the distribution of change in these variables during follow-up. Figure 3 shows ACE-R and mRS score interrelationships at each visit according to WMH change quintiles.

eTable 4 gives mean WMH volumes, cognitive score, and mRS score.

### Scores at Each Time Point

Between baseline and 1- or 3-year follow-up, 95 of 195 (48.7%) participants had a ≥1-point increase in mRS score, and 76 of 148 (51.3%) had a ≥1-point decrease in ACE-R score. Considered together, 28 of 137 (20.4%) participants had a combined increase in mRS score with decrease in ACE-R score, potentially meeting DSM-V diagnostic criteria for dementia.

### Multivariable Analyses

#### Cross-sectional WMH, Cognition Score, and mRS Score at Baseline and 1 and 3 Years

**Figure 4:**
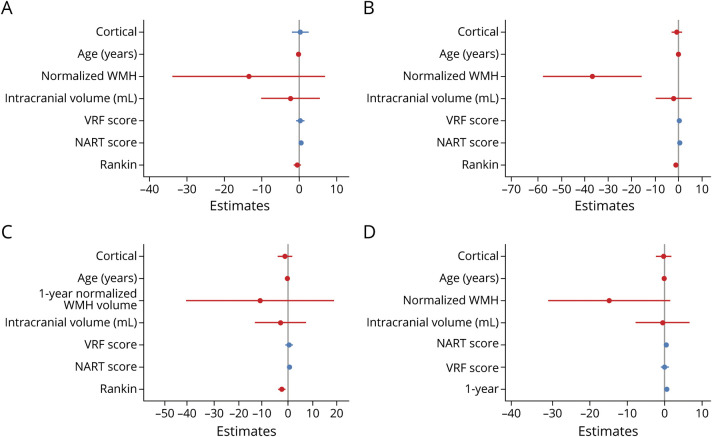
Cross-Sectional and Longitudinal Associations With ACE-R Scores (A-C) Linear models: Addenbrooke's Cognitive Assessment–Revised (ACE-R) associations at (A) baseline, (B) 1 year, and (C) 3 years. (D) Mixed-effects model: intraindividual changing ACE-R associations between baseline and 1 year. Note that we used 1-year white matter hyperintensity (WMH) volumes in panel C. NART = National Adult Reading Test; VRF = vascular risk factor.

First, we performed cross-sectional analyses of ACE-R score associations at baseline, 1-year, and 3-year visits (eTable 5, links.lww.com/WNL/B796 and Figure 4, A–C). At baseline, ACE-R scores were very weakly associated with WMH volumes (cube root as percent ICV) (β = 0.105, 95% CI −0.265 to 0.054 more WMH per 1-point ACE-R score decrease, *p* = 0.195). At 1 year, associations between ACE-R scores and WMH volumes were more apparent (β = −0.259, 95% CI −0.407 to −0.111 more WMH per 1-point ACE-R score decrease, *p* = 0.001). The ACE-R/WMH association predominated over ACE-R score associations with age, mRS score, ICV, vascular risk factors, and stroke subtype. Because participants did not undergo imaging at 3 years, we included normalized WMH volumes from the 1-year visit in the 3-year model. We did not detect any associations between 1-year WMH and 3-year ACE-R scores. Assessing ACE-R and mRS score relationships, we did not find any association between ACE-R and mRS scores at baseline. However, we detected an ACE-R/mRS score trend at 1 year, and a strong association emerged at 3 years (β = −0.272, 95% CI −0.429 to −0.115, *p* = 0.001; eTable 5 and Figure 4, A–C).

We then ran cross-sectional ordinal regression models of mRS score associations at baseline and 1 and 3 years (eTable 6). We used 1-year WMH volumes and NIHSS scores in the 3-year model. WMH were not associated with mRS score at any time point (1-year odds ratio [OR] 1.01, 95% CI 0.99–1.02). NIHSS score was contemporaneously associated with mRS score at baseline (OR 4.19, 95% CI 2.89–6.25), 1 year (OR 4.28, 95% CI 2.89–6.25), and 3 years (OR 3.08, 95% CI 2.51–7.59).

#### Longitudinal WMH, Cognition Score, and mRS Score Between Baseline and 1 Year

Second, we assessed longitudinal change according to intraindividual trajectories. We used mixed-effects models to assess ACE-R score, mRS score, and WMH volumes at baseline and 1 year. Changing ACE-R scores were associated most strongly with age (β = −0.30, 95% CI −0.44 to −0.16, *p* < 0.001) and NART scores (β = 0.53, 95% CI 0.40–0.66, *p* < 0.001), followed by change in normalized WMH volumes between baseline and 1 year (β = −0.113, 95% CI −0.233 to 0.007, *p* = 0.065; Figure 4D and eTable 7, links.lww.com/WNL/B796). ACE-R score change was more strongly associated with WMH volume change than with mRS score change, time since stroke, ICV, vascular risk factors, and stroke subtype. Change in mRS score from baseline to 1 year was associated most strongly with NIHSS score and WMH volumes, with a trend toward cortical (rather than lacunar) stroke subtypes (eTable 8).

#### Longitudinal WMH and Combined, Covarying Cognitive/mRS Score Endpoint Between Baseline and 1 Year

Last, in multivariate change-change analysis, individuals with change in both ACE-R and mRS scores between baseline and 1 year were more likely to have had a baseline-1-year change in their WMH volumes and a baseline–1-year change in their NIHSS scores compared with individuals without any change in ACE-R and mRS scores (Table 2). Worsening ACE-R/mRS scores were also more likely in participants with lower ICV and participants who were older at baseline. Covariation between ACE-R and mRS scores was stronger at the 1-year visit than at the baseline visit.

**Table 2:**
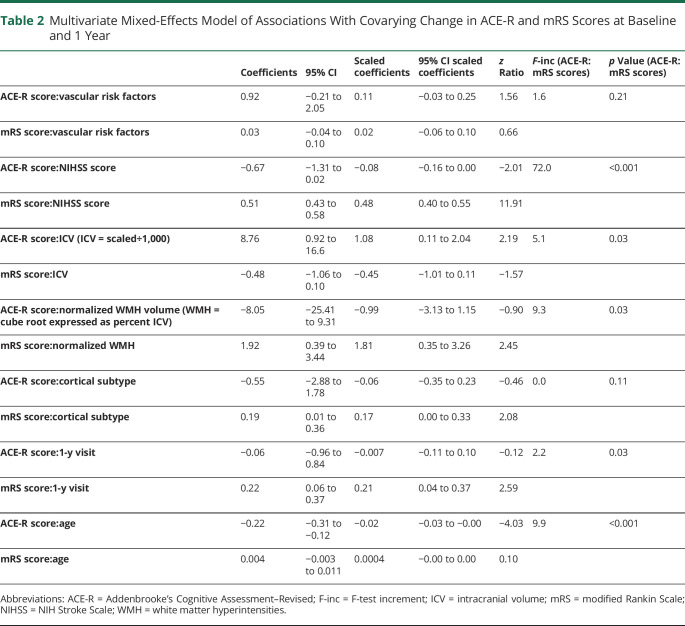
Multivariate Mixed-Effects Model of Associations With Covarying Change in ACE-R and mRS Scores at Baseline and 1 Year

Table 2 shows the contribution of each variable driving ACE-R score change and mRS score change between baseline and 1 year; for example, change between baseline–1-year WMH volumes and covarying ACE-R/mRS scores during the same time period is driven strongly by WMH:mRS score (standardized mRS score change 1.81, 95% CI 0.35–3.26) than by WMH:ACE-R score (standardized ACE-R score change −0.99, 95% CI −3.13 to 1.15]). In contrast, covarying ACE-R/mRS score change associations with baseline ICV and age are more strongly associated with changing ACE-R scorer than with changing mRS score.

## Discussion

We found that covarying longitudinal change in cognition and mRS scores after stroke independently is associated with change in total WMH volumes between baseline and 1 year. In the subacute phase after stroke, cross-sectional cognitive scores are most strongly associated with age and premorbid cognitive ability (NART score), followed by WMH volume. However, by 1 year, the WMH-cognition cross-sectional association is considerably stronger and supersedes all other factors (Figure 4).

Longitudinal change in cognitive scores is associated most strongly with age and NART score, followed by change in WMH volumes, then mRS score, baseline ICV, baseline vascular risk factors, stroke subtype, and timing (baseline vs 1 year) since stroke. A WMH–mRS score association emerges over time that was not detectable on cross-sectional analyses, reflecting that WMH and mRS score are dynamic and effects may take longer to emerge and stabilize.

Of particular relevance to dementia after stroke, we show that longitudinal change in covarying cognition and mRS score in the first year after stroke is associated with age, ICV, change in WMH volumes, and NIHSS score.

These findings highlight the role of SVD in evolving vascular cognitive impairment and physical dysfunction after stroke, suggesting that individuals with WMH progression after stroke are more likely to develop a simultaneous decline in cognition and mRS score compared to individuals with unchanging WMH after accounting for demographics and risk factors. This analysis builds on the recent finding in the same population that cognitive impairment after lacunar stroke is related more to background SVD than to index lesion location.^35^ That finding notwithstanding, considering the range of all subtypes of stroke, lesion location does appear to play a role in poststroke cognitive impairment.^36^ These findings add clarity to the time course of vascular cognitive impairment and the impact of WMH progression in the first year after stroke, both increases and decreases, suggesting that clinical evidence of SVD-related cognitive or mRS score change becomes progressively apparent in the chronic rather than subacute phase after stroke (Figure 3).

Our finding of associations between cognitive decline and WMH progression was not detected in most previous longitudinal imaging studies after stroke.^12-14,16,17^ These differences may be due to variation in follow-up sample sizes, cognitive measures, methods of assessing WMH change, lower dropout rates, and follow-ups with the same vs separate scanners. Moreover, the interval between stroke presentation and baseline MRI assessment is an important factor (eTable 2, links.lww.com/WNL/B796) because SVD lesions are dynamic.^1^ In the assessment of poststroke cognition, it is important to adjust for factors that are associated with premorbid cognitive ability,^22^ later-life cerebrovascular disease,^41,42^ and cognitive decline.^42^ One previous study adjusted for ICV and education,^12^ and another adjusted for premorbid IQ.^37^

Apart from WMH, a broader range of SVD features require further longitudinal attention in relation to combined cognitive decline^37^ and worsening mRS score after stroke. Such features, including lacunes, perivascular spaces, and microbleeds, are part of the total SVD picture.^25^ Total baseline SVD scores are associated with lower cognitive ability in older adults^43^ and with worse prognosis in patients with stroke.^44^ Further work is required to establish how total longitudinal SVD changes might relate to clinical outcomes.

This study uses a statistical approach combining cognition and mRS score as a coprimary outcome, paralleling real-world DSM-V diagnostic criteria^7^ for the clinical diagnosis of dementia. We used a well-validated approach to quantify WMH volumes at 2 time points with improved retention (n = 196 of 264) for follow-up MRI compared with previous longitudinal SVD studies assessing cognition after stroke (n = 94 of 115 at 1 year and 74 of 115 at 2 years^14,37^; n = 52 of 101,^12^ n = 101 of 189 [CT based],^38^ n = 52 of 81 at 5 years^17^) and compared with nonstroke longitudinal MRI studies.^5,39,40^

Our study had limitations. We did not invite participants to attend MRI at 3 years; longer-term WMH volumes would have strengthened our analysis and better reflected the dynamic changes of WMH, cognition score, and mRS score at all 3 time points. Not all participants completed cognitive assessments (eTable 3, links.lww.com/WNL/B796) because we introduced cognitive tests after the first patients had joined the study, but completion rates for mRS were good (84% at 3 years; eTable 3). We did not include depression as a covariate: depression is common after stroke, has associations with worse WMH, and has implications for cognitive dysfunction and dependence.

This study found that the key components of the dementia syndrome, combined decline in cognition and independence, are associated with the presence and progression of WMH in the year after a stroke. One-year, but not baseline, WMH volumes are strongly associated with contemporaneous cognitive scores, superseding other clinical factors. This suggests that dynamic WMH in the weeks after a stroke do not reflect permanent brain damage, but by 1 year, perhaps when any modifiable component of WMH such as interstitial oedema has cleared,^45,46^ WMH volumes may more closely represent the underlying permanent damage and hence better correlate with cognition. Studies evaluating patients for as long as possible after stroke, that is, to 3 years and beyond, are needed to capture the full long-term implications.

These results call for an acceleration of research into processes driving SVD-related cognitive decline. The emergence of a WMH-cognitive association 1 year after a stroke represents a short but achievable therapeutic window for halting SVD progression and potentially preserving cognitive ability and independence after stroke.

It would be useful to determine whether a combined, covarying cognitive and mRS score outcome could predict conversion to incident dementia in the longer term. We should encourage consistent analysis of levels of independence alongside cognitive ability in stroke research. A more detailed spatial analysis assessing patterns with persisting WMH vs WMH that dynamically appear and disappear would give more precise insights into the influence of SVD on the clinical course after a stroke. Moreover, an analysis of whether WMH lesions behave differently adjacent to vs remote from the stroke lesion, whether this affects lesion evolution, and whether this influences outcomes after stroke is required.

Despite secondary prevention, vascular risk factors were not associated with change in cognitive scores in our study. This reinforces the finding that alternative treatment approaches to target the underlying pathophysiology of SVD are particularly important.

We need to closely track the natural history of dementia after stroke and to determine whether clinically and radiologically distinct dementia subtypes emerge over time. Identifying subgroups will allow future triage of clinical presentations to appropriate services, the development of disease-specific management strategies, and targeted entry into future research trials.

## Glossary

ACE-R: Addenbrooke’s Cognitive Examination–Revised
DSM-V: *Diagnostic and Statistical Manual of Mental Disorders, 5th edition*
FLAIR: fluid-attenuated inversion recovery
ICV: intracranial volume
IQR: interquartile range
mRS: modified Rankin Scale
NART: National Adult Reading Test
NIHSS: NIH Stroke Scale
OR: odds ratio
SVD: small vessel disease
WMH: white matter hyperintensities
